# Supplementary figures and images for: TORC1-dependent translation drives chromatin remodeling during the germ-cell-to-maternal transition in Drosophila
Source: EMBO J. 2026 Jan 26;45(5):1648–71. doi: 10.1038/s44318-026-00697-0 (PMC12953698; doi:10.1038/s44318-026-00697-0)

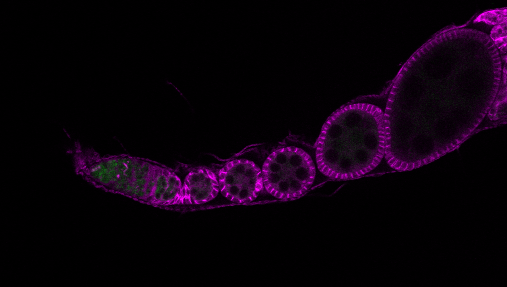

Supplement: Supplementary file 6 — Source data Fig. 1 [file 44318_2026_697_MOESM6_ESM.zip › Figure 1/1C/rps19b-gfp control.tif]

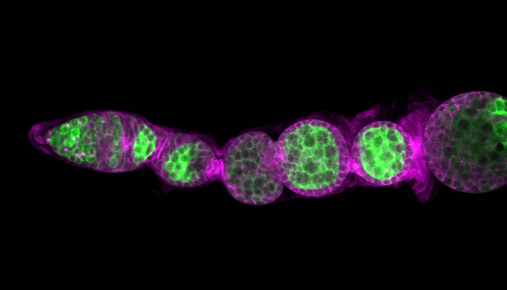

Supplement: Supplementary file 6 — Source data Fig. 1 [file 44318_2026_697_MOESM6_ESM.zip › Figure 1/1C/rps19b x mio RNAi.tif]

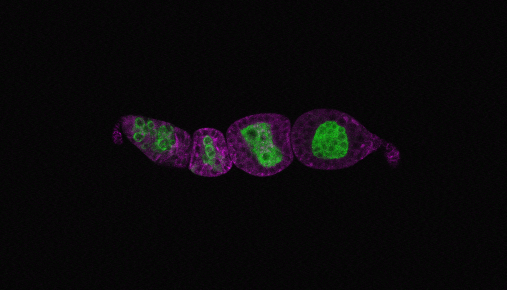

Supplement: Supplementary file 6 — Source data Fig. 1 [file 44318_2026_697_MOESM6_ESM.zip › Figure 1/1C/rps19bGFP x eEF1a1 RNAi.tif]

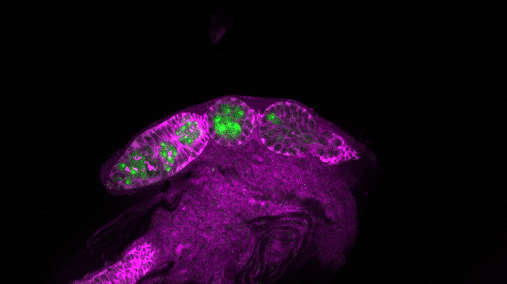

Supplement: Supplementary file 6 — Source data Fig. 1 [file 44318_2026_697_MOESM6_ESM.zip › Figure 1/1C/rps19bGFP x aramis RNAi.tif]

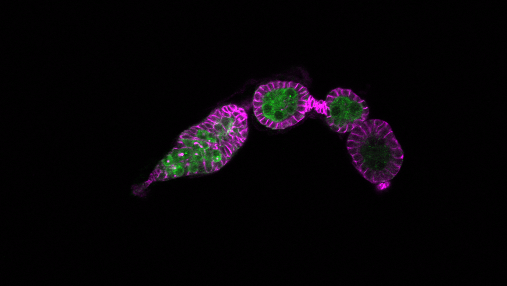

Supplement: Supplementary file 6 — Source data Fig. 1 [file 44318_2026_697_MOESM6_ESM.zip › Figure 1/1C/rps19bgfp x bystin RNAi.tif]

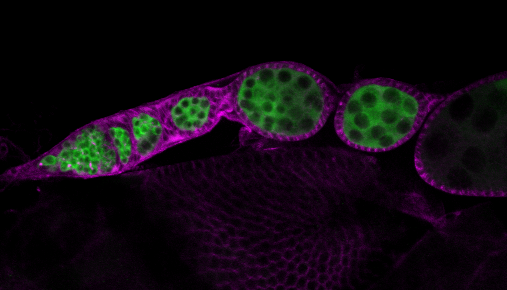

Supplement: Supplementary file 6 — Source data Fig. 1 [file 44318_2026_697_MOESM6_ESM.zip › Figure 1/1C/rps19b x raptor RNAi.tif]

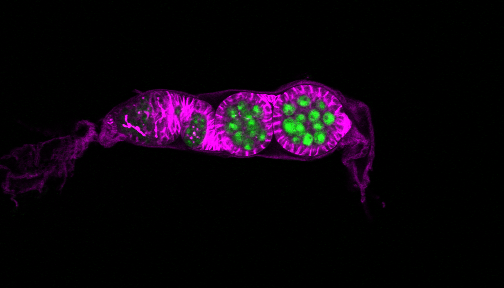

Supplement: Supplementary file 6 — Source data Fig. 1 [file 44318_2026_697_MOESM6_ESM.zip › Figure 1/1C/rps19bGFP x zfrp8 RNAi.tif]

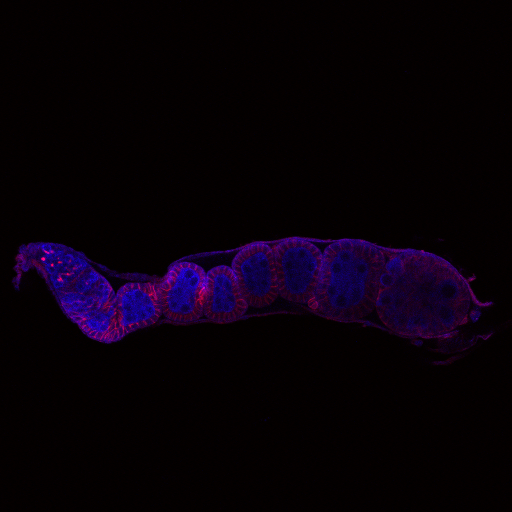

Supplement: Supplementary file 9 — Source data Fig. 4 [file 44318_2026_697_MOESM9_ESM.zip › Figure 4/4E/UAS Dicer 2 NG4 x lolal RNAi.tif]

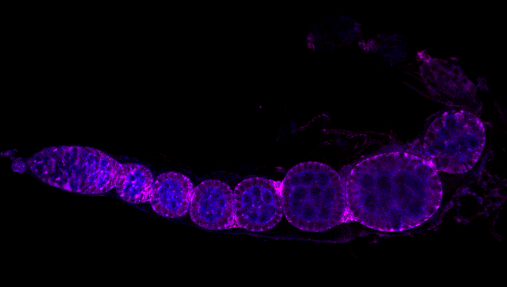

Supplement: Supplementary file 9 — Source data Fig. 4 [file 44318_2026_697_MOESM9_ESM.zip › Figure 4/4E/UAS Dicer 2 NG4 x nup44a RNAi.tif]

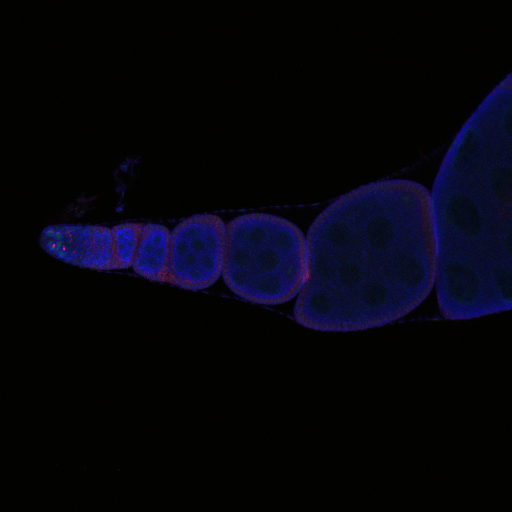

Supplement: Supplementary file 9 — Source data Fig. 4 [file 44318_2026_697_MOESM9_ESM.zip › Figure 4/4E/UAS Dicer2 NG4 control.tif]

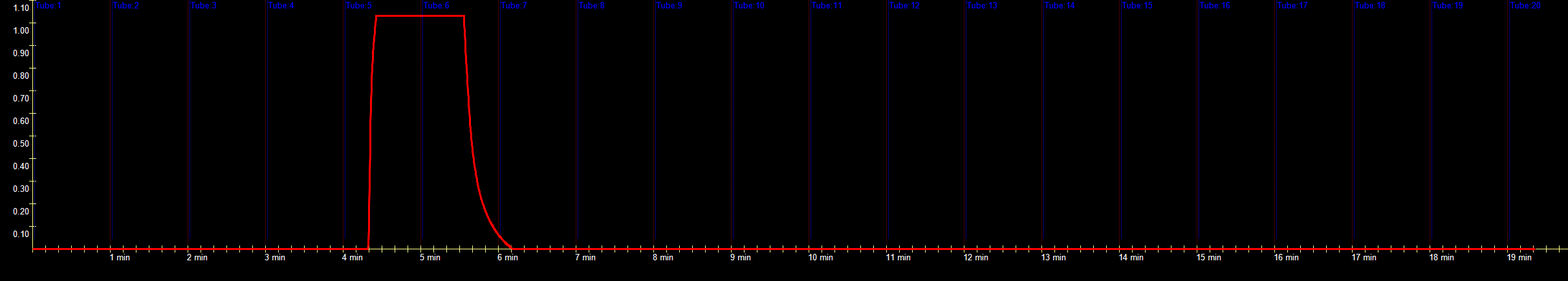

Supplement: Supplementary file 9 — Source data Fig. 4 [file 44318_2026_697_MOESM9_ESM.zip › Figure 4/4B/Wildtype 1/Chart.bmp]

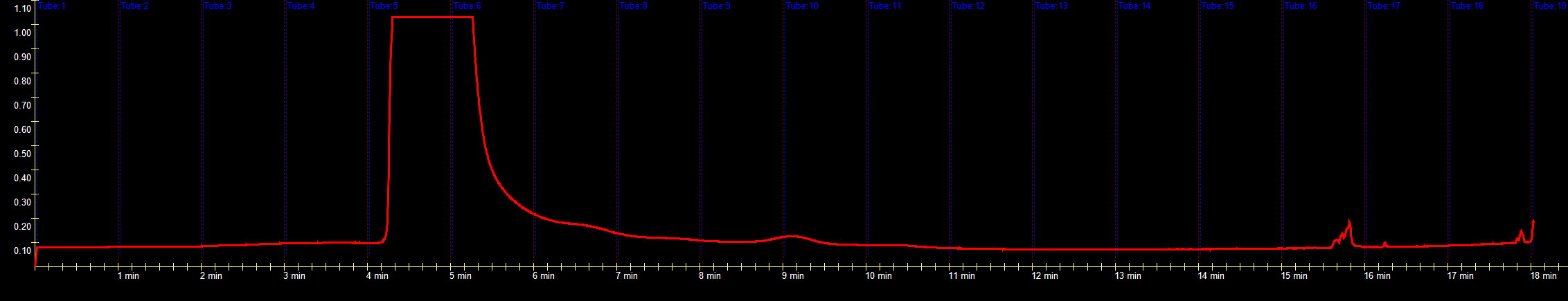

Supplement: Supplementary file 9 — Source data Fig. 4 [file 44318_2026_697_MOESM9_ESM.zip › Figure 4/4B/Zfrp8 GKD 1/Chart.bmp]

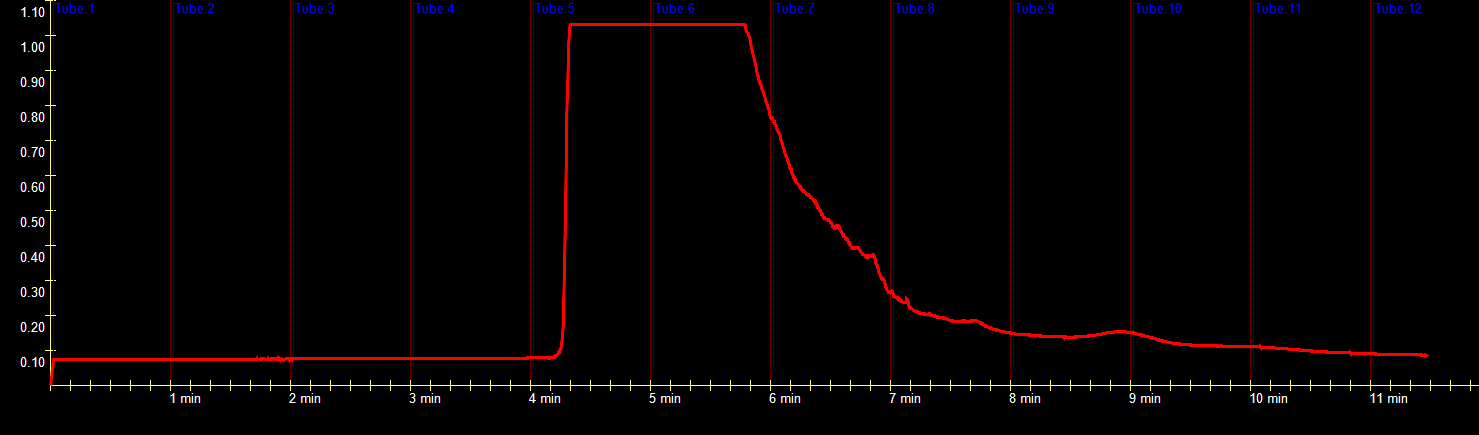

Supplement: Supplementary file 9 — Source data Fig. 4 [file 44318_2026_697_MOESM9_ESM.zip › Figure 4/4B/Zfrp8 GKD 3/Chart.bmp]

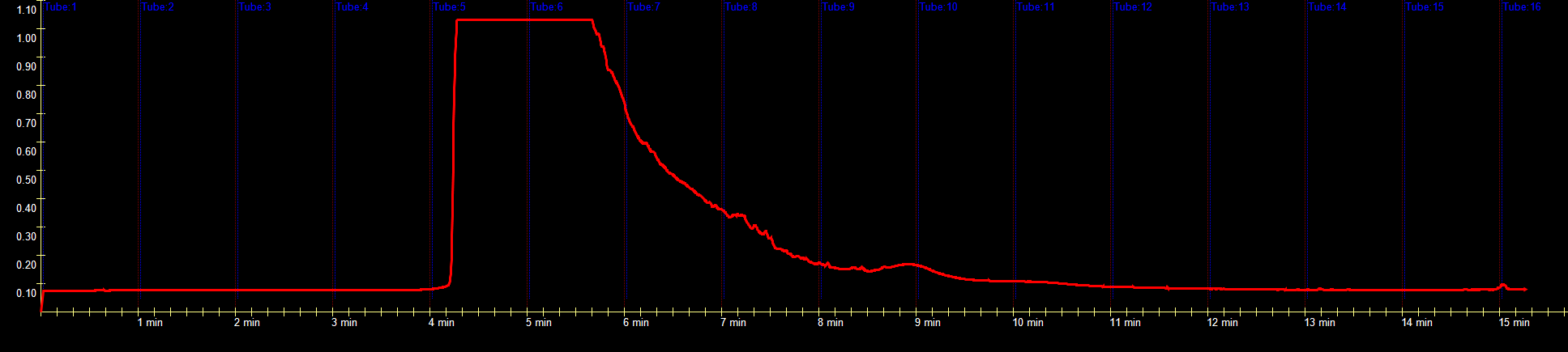

Supplement: Supplementary file 9 — Source data Fig. 4 [file 44318_2026_697_MOESM9_ESM.zip › Figure 4/4B/Zfrp8 GKD 2/Chart.bmp]

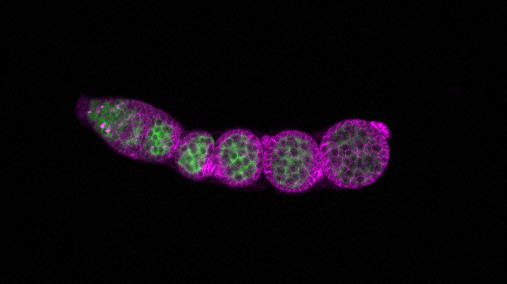

Supplement: Supplementary file 10 — Source data Fig. 5 [file 44318_2026_697_MOESM10_ESM.zip › Figure 5/5A/rps19b-NG4 x nup44a RNAi.tif]

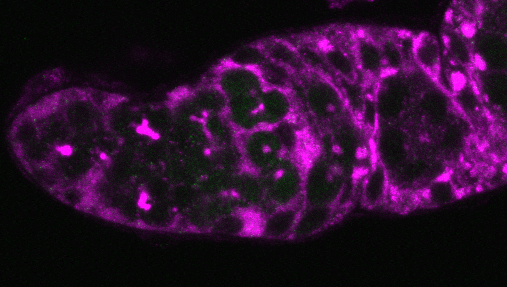

Supplement: Supplementary file 11 — Source data Fig. 6 [file 44318_2026_697_MOESM11_ESM.zip › Figure 6/6B/TOP rep x mio RNAi.tif]

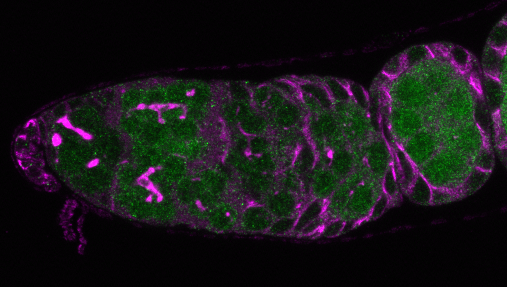

Supplement: Supplementary file 11 — Source data Fig. 6 [file 44318_2026_697_MOESM11_ESM.zip › Figure 6/6B/TOP rep control.tif]

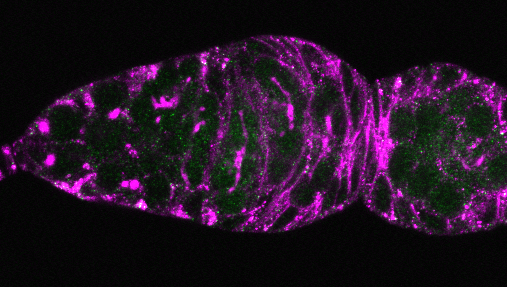

Supplement: Supplementary file 11 — Source data Fig. 6 [file 44318_2026_697_MOESM11_ESM.zip › Figure 6/6B/TOP rep x nup44a RNAi.tif]

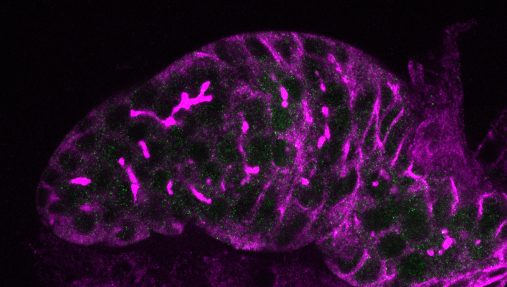

Supplement: Supplementary file 11 — Source data Fig. 6 [file 44318_2026_697_MOESM11_ESM.zip › Figure 6/6B/TOP rep x zfrp8 RNAi.tif]

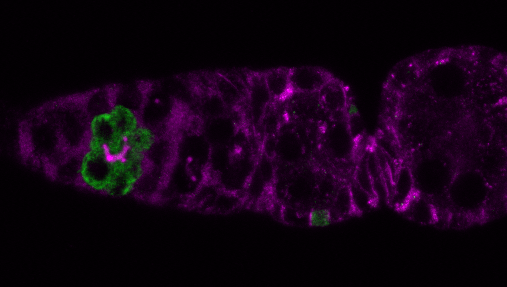

Supplement: Supplementary file 12 — Expanded view and appendix Source Data [file 44318_2026_697_MOESM12_ESM.zip › Source data for expanded view and appendix/Extended View Figure 1/EV1C/p-s6 in Dcr2_40X cropped_7_ps.tif]

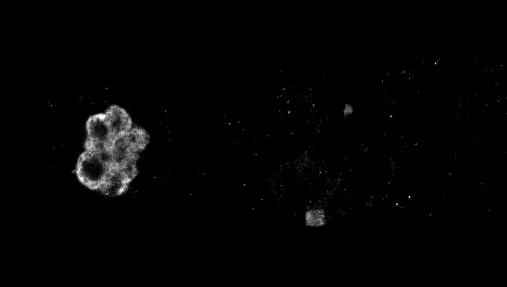

Supplement: Supplementary file 12 — Expanded view and appendix Source Data [file 44318_2026_697_MOESM12_ESM.zip › Source data for expanded view and appendix/Extended View Figure 1/EV1C/p-s6 in Dcr2_40X cropped_7_red1b1_ps_ps6.tif]

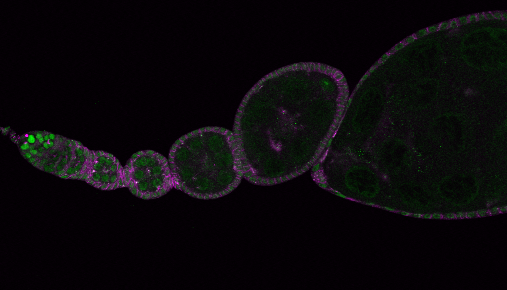

Supplement: Supplementary file 12 — Expanded view and appendix Source Data [file 44318_2026_697_MOESM12_ESM.zip › Source data for expanded view and appendix/Extended View Figure 1/EV1D/bamGAL4 blanks control.tif]

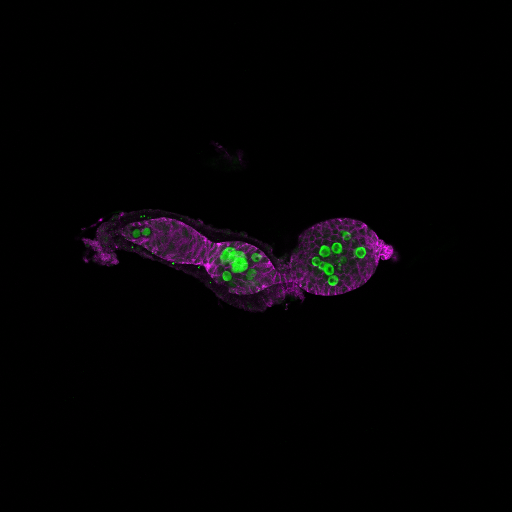

Supplement: Supplementary file 12 — Expanded view and appendix Source Data [file 44318_2026_697_MOESM12_ESM.zip › Source data for expanded view and appendix/Extended View Figure 1/EV1D/bamGAL4 x mio RNAi blanks.tif]

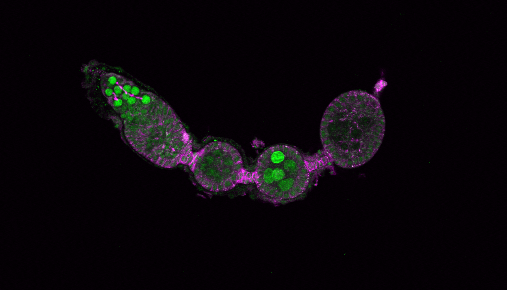

Supplement: Supplementary file 12 — Expanded view and appendix Source Data [file 44318_2026_697_MOESM12_ESM.zip › Source data for expanded view and appendix/Extended View Figure 1/EV1D/bamGAL4 x zfrp8 RNAi blanks.tif]

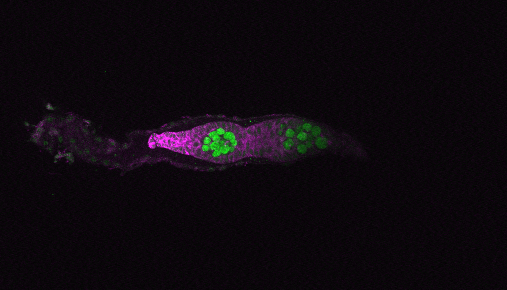

Supplement: Supplementary file 12 — Expanded view and appendix Source Data [file 44318_2026_697_MOESM12_ESM.zip › Source data for expanded view and appendix/Extended View Figure 1/EV1D/bamGAL4 x eEF1a1 blanks.tif]
