# Supplementary material for: TORC1-dependent translation drives chromatin remodeling during the germ-cell-to-maternal transition in Drosophila
Source: EMBO J. 2026 Jan 26;45(5):1648–71. doi: 10.1038/s44318-026-00697-0 (PMC12953698; doi:10.1038/s44318-026-00697-0)
Supplement: Supplementary file 13 — Expanded View Figures [file 44318_2026_697_MOESM13_ESM.pdf]

Expanded View Figures

Figure EV1. Ribosome-level regulators are required in the cyst stages for silencing germ cell gene *blanks* during oogenesis.

(**A**) Quantification of the size of the egg chambers of control and *zfrp8* GKD, *bystin* GKD, *mio* GKD, *raptor* GKD and *eEF1a1* GKD ovaries. Graph shows that *zfrp8* GKD, *bystin* GKD, *mio* GKD, and *raptor* GKD's egg chambers do not grow compared to control ovaries. *eEF1a1* GKD did not lead to the formation of a third egg chamber therefore we were unable to quantitate. Statistics: Two-tailed *t* test; $n = 5$ ovarioles per genotype; $P = 0.0015$ for *aramis* GKD; $P = 0.0011$ for *zfrp8* GKD; $P = 0.0233$ for *mio* GKD; $P = 0.0010$ for *bystin* GKD; $P = 0.0138$ for *raptor* GKD. (**B**) Arbitrary unit (A.U.) quantification of RpS19b::GFP expression in *zfrp8* GKD, *bystin* GKD, *mio*GKD, *raptor* GKD and *eEF1a1* GKD compared to control ovaries. RpS19b::GFP persists in egg chambers of *zfrp8*GKD, *bystin* GKD, *mio* GKD, *raptor* GKD and *eEF1a1* GKD compared to the egg chambers of control. $n = 5$ ovarioles per genotype. Statistics: Two-tailed *t* test; $n = 50$ ovarioles per genotype; $P < 0.0001$ for *aramis* GKD $P < 0.0001$ for *zfrp8* GKD; $P < 0.0001$ for *mio* GKD; $P < 0.0001$ for *bystin* GKD; $P < 0.0001$ for *raptor* GKD; $P < 0.0001$ for *eEF1a1* GKD. (**C**) Wild-type control germarium stained for p-S6 (green, right in grayscale) and 1B1 (magenta). p-S6 marking TOR activity is expressed in the cyst stages. (**D**) Ovarioles stained for Blanks (green, shown in grayscale on right) and 1B1 (magenta). In *bamGAL4* controls, Blanks is expressed in undifferentiated germ cells (yellow arrows) and attenuated in egg chambers. In contrast, GKD of *zfrp8*, *mio*, or *eEF1a1* using *bamGAL4* caused egg chambers to ectopically express Blanks (white dashed lines), fail to grow, and degenerate during oogenesis. (**E**) Quantitation of percent ovarioles with Blanks expansion in control, *zfrp8* GKD, *bystin* GKD, *mio* GKD, *raptor* GKD and *eEF1a1* GKD ovaries. Statistics: Fisher's exact test; $n = 50$ ovarioles per genotype; $P < 0.0001$ for *zfrp8* GKD; $p < 0.0001$ for *mio* GKD; $P < 0.0001$ for *eEF1a1* GKD. Scale bars: (C-C1) 7.5 µm; (D-G1) 15 µm. Source data are available online for this figure.

 © The Author(s)

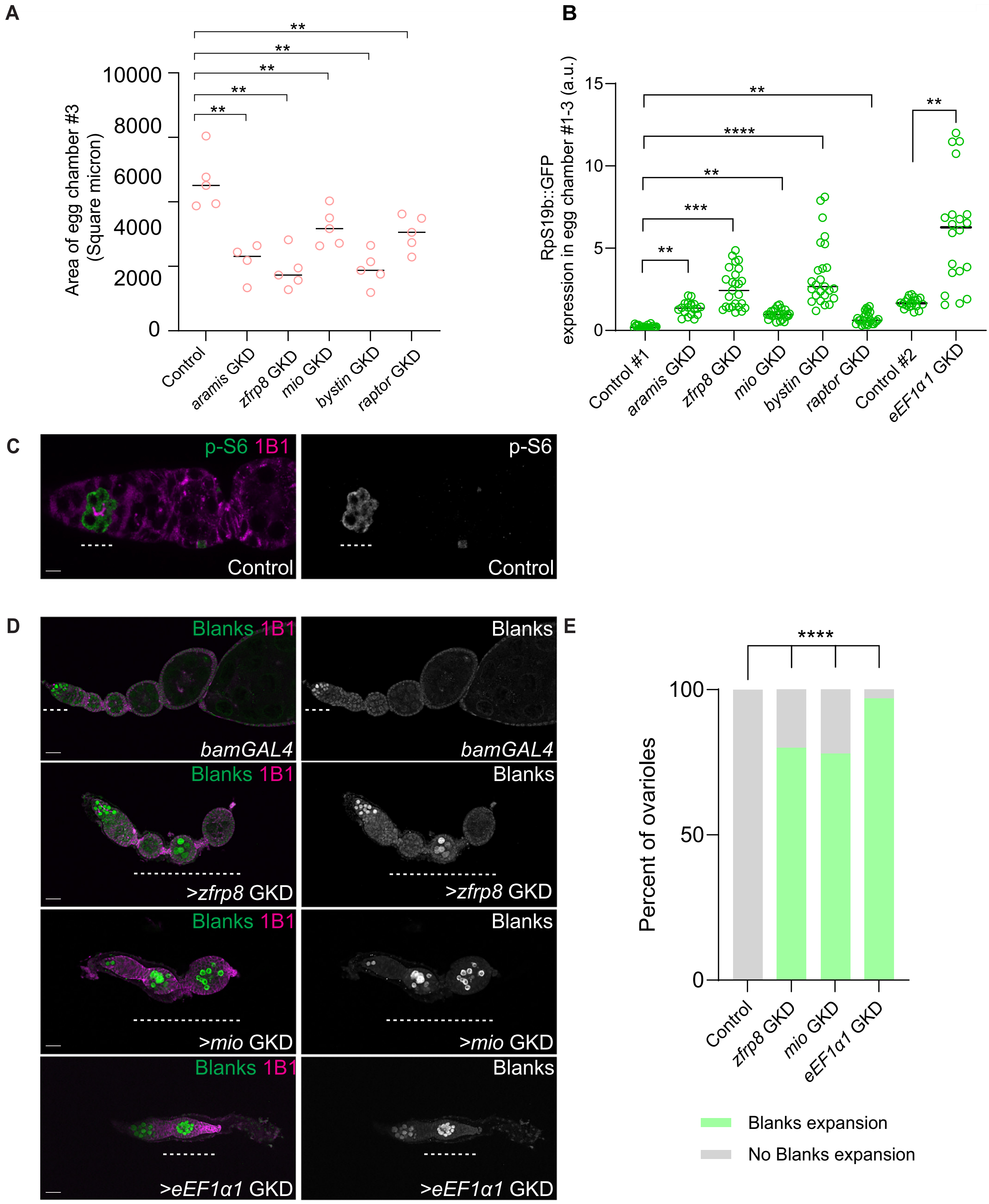
A
Area of egg chamber #3
(Square micron)
10000
8000
6000
4000
2000
0
**
Control
aramis GKD
zfrp8 GKD
mio GKD
bystin GKD
raptor GKD
B
RpS19b::GFP
expression in egg chamber #1-3 (a.u.)
15
10
5
0
**
***
****
Control #1
aramis GKD
zfrp8 GKD
mio GKD
bystin GKD
raptor GKD
Control #2
eEF1α1 GKD
C
p-S6 1B1
p-S6
Control
D
Blanks 1B1
Blanks
bamGAL4
>zfrp8 GKD
>mio GKD
>eEF1α1 GKD
E
Percent of ovarioles
100
50
0
****
Control
zfrp8 GKD
mio GKD
eEF1α1 GKD
Blanks expansion
No Blanks expansion

© The Author(s)

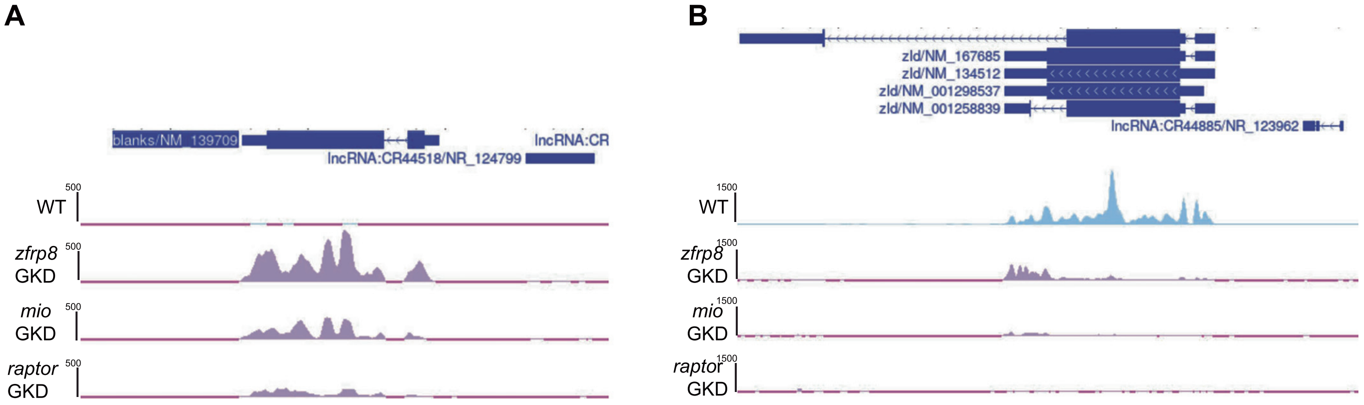

Figure EV2. Ribosome-level regulators are required for silencing germ cell genes during oogenesis.

(**A**) RNA-seq tracks showing that *blanks* is upregulated upon >*zfrp8* GKD, >*mio* GKD and >*raptor* GKD (purple) compared to control *nosGAL4* (blue). (**B**) RPKM-normalized RNA-seq tracks showing that *zelda* is downregulated upon >*zfrp8* GKD, >*mio* GKD and >*raptor* GKD (purple) compared to control (*nosGAL4)* (blue).

© The Author(s)

A

B

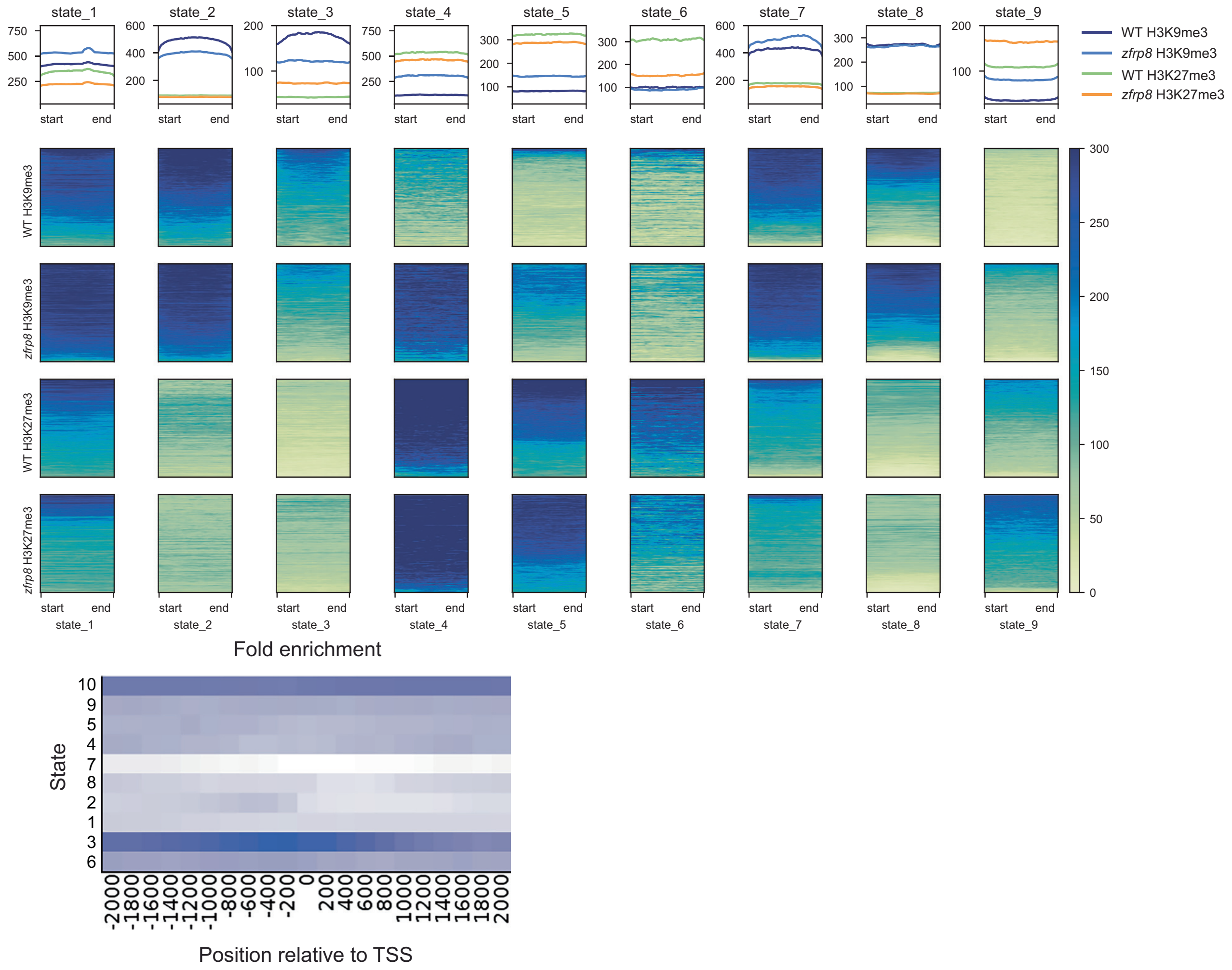

Figure EV3. Ribosome-level regulators are required maintaining proper chromatin state.

(**A**) A 10-state chromatin model depicting H3K9me3 and H3K27me3 distribution across the genome. This model illustrates H3K9me3 and H3K27me3 dynamics across 10 chromatin states, highlighting their distribution in different genomic regions. In *zfrp8* GKD, H3K9me3 is reduced on promoters in state 3. These findings reveal that ribosome biogenesis influences genome-wide epigenetic landscapes, coordinating transcriptional regulation during oogenesis. (**B**) In state 3, H3K9me3 is enriched around promoters. Source data are available online for this figure.

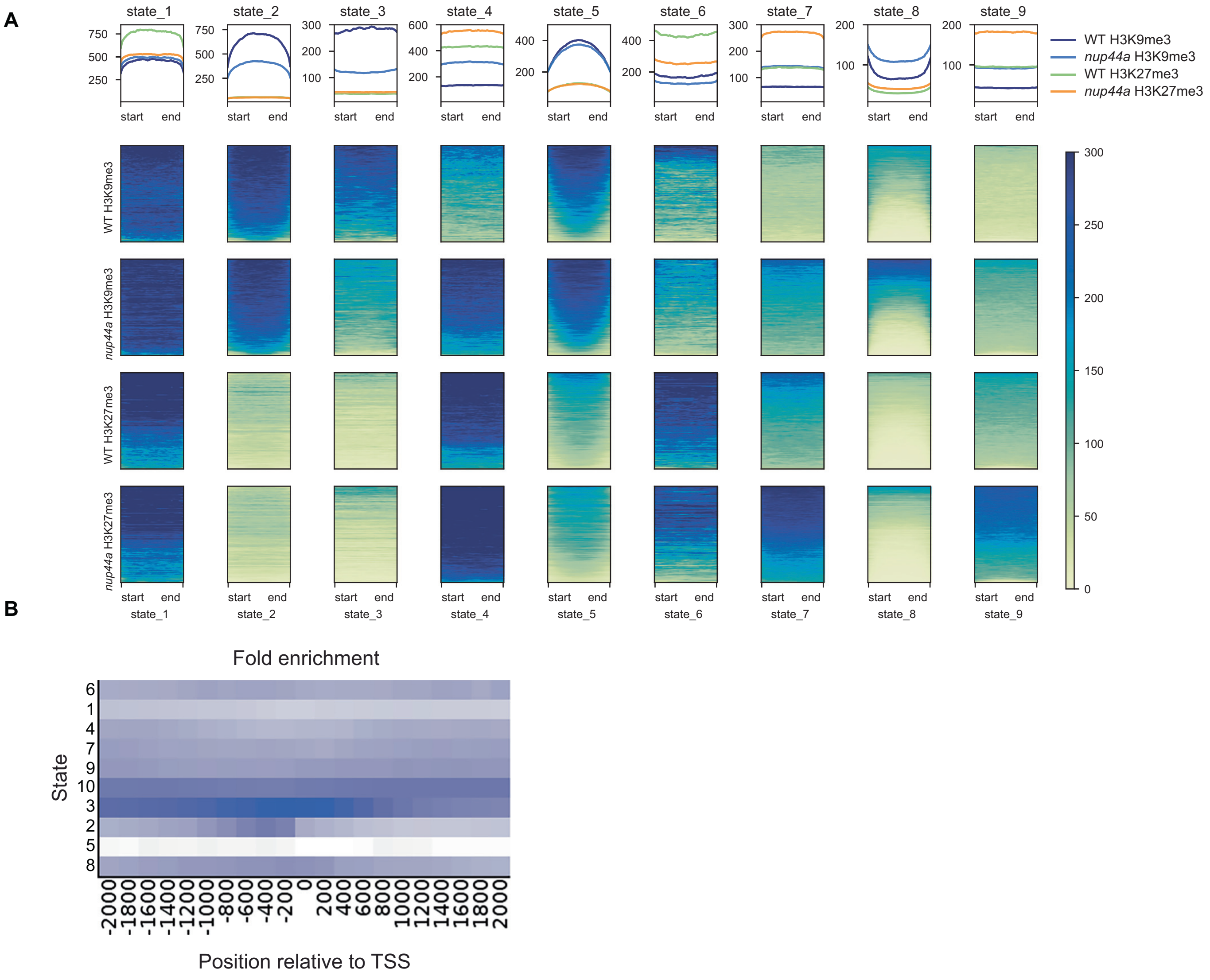

Figure EV4. Ribosome-level regulators are required for maintaining proper chromatin state.

(**A**) A 10-state chromatin model depicting H3K9me3 and H3K27me3 distribution across the genome. This model illustrates H3K9me3 and H3K27me3 dynamics across 10 chromatin states, highlighting their distribution in different genomic regions. In *Nup44A* GKD, H3K9me3 is reduced on promoters in State 3 and H3K27me3 is redistributed across different states, indicating widespread chromatin reorganization. These findings reveal that Nup44A influences genome-wide epigenetic landscape. (**B**) In state 3, H3K9me3 is enriched around promoters, ensuring transcriptional repression of genes including *rps19b*. Source data are available online for this figure.

 © The Author(s)
